# Evaluation of gemcitabine efficacy after the FOLFIRINOX regimen in patients with advanced pancreatic adenocarcinoma

**DOI:** 10.1097/MD.0000000000006544

**Published:** 2017-04-21

**Authors:** Marine Gilabert, Brice Chanez, Young Soo Rho, Marc Giovanini, Olivier Turrini, Gerald Batist, Petr Kavan, Jean Luc Raoul

**Affiliations:** a Department of Medical Oncology, Paoli-Calmettes Institute, Marseille, France; b Department of Medical Oncology, Jewish General Hospital, McGill University, Montréal, QC , Canada; c Department of Gastroenterology; d Department of Digestive Surgery, Paoli-Calmettes Institute, Marseille, France.

**Keywords:** chemotherapy toxicities, FOLFIRINOX regimen, gemcitabine efficacy, pancreatic adenocarcinoma, survival study

## Abstract

To evaluate gemcitabine efficacy in advanced pancreatic cancer patients after the FOLFIRINOX regimen.

Patients with locally-advanced or metastatic pancreatic adenocarcinoma from French and Canadian centers, who were treated with the first-line FOLFIRINOX regimen (FFX L1), followed by gemcitabine monotherapy as a second-line treatment (GEM L2), were retrospectively evaluated. Statistical analyses were performed on the demographic, toxicity, and response rate data. Overall survival (OS) and progression-free survival (PFS) were assessed using the Kaplan–Meier method.

Seventy-two patients were reviewed (median age of 63.5 years [range, 32–75 years], men [62%], predominantly pancreatic head tumor location [51%] and metastatic disease [64%] at the time of diagnosis). The objective response rate to GEM-L2 treatment was 8/72 (11%), and 32 patients (44%) experienced a clinical benefit from gemcitabine. Four patients had a partial response to GEM-L2, although they previously showed a progressive response following FFX-L1 treatment. The median OS for the entire cohort was 13.6 months (95% confidence interval [CI]: 2.0–35). The median PFS of the GEM-L2 group was 2.5 months (95% CI: 0.2–10.8) with no statistical differences between patients with controlled or progressive disease on FFX-L1 therapy.

Gemcitabine as a second-line treatment for advanced pancreatic adenocarcinoma after FOLFIRINOX failure showed clinical benefits in some patients.

## 1 Introduction

Pancreatic ductal adenocarcinoma (PDAC) is one of leading cause of cancer deaths among men and women worldwide, and it is responsible for 6% of all cancer deaths.^[1]^ Despite decades of effort and in contrast with improved survival for many cancers, PDAC life expectancy remains poor, with a 5-year survival of 5% to 10%.^[2]^ Treatment of this disease is based on a multidisciplinary approach that includes surgery, radiotherapy, and chemotherapy, although the impact of therapy in metastatic cases is only palliative. In 1997, a randomized trial in patients with advanced symptomatic pancreatic cancer compared gemcitabine with fluoro-uracil (5FU). Gemcitabine monotherapy resulted in a longer survival compared with that of the 5FU arm, and it was approved as a standard care in this setting.^[3]^ Since that trial, many clinical trials have combined cytotoxic drugs or biotherapies with gemcitabine, but no significant improvements in outcome were observed. A significant improvement in survival was achieved in 2010 with the FOLFIRINOX trial.^[4]^ Patients treated with a combination of 5FU, oxaliplatin, and irinotecan showed significantly longer overall survival (OS) compared with that of patients in the gemcitabine monotherapy arm, with a median OS of 11.1 months versus 6.8 months, and this occurred without a decrease in the quality of life. This new treatment paradigm was internationally approved in 2011 as a first-line treatment for advanced metastatic PDAC. Since then, single agent gemcitabine is no longer the preferred first-line chemotherapy treatment, except for elderly or deteriorated patients, and many physicians use it as a second-line treatment. However, there is no clear recommendation from the National Comprehensive Cancer Network (NCCN) for using gemcitabine as a second-line therapy and few reports are available in the literature that evaluate the efficacy and response rates of gemcitabine after a FOLFIRINOX regimen. The goal of our retrospective study was to evaluate gemcitabine monotherapy efficacy, response rates, and toxicity profile when used as a second-line treatment in advanced pancreatic cancer patients previously treated by a FOLFIRINOX regimen.

## 2 Patients and methods

### 2.1 Patients

We retrospectively analyzed patients treated for locally-advanced or metastatic pancreatic adenocarcinoma at the Paoli-Calmettes Institute, Marseille, France and at the Segal Cancer Center, McGill University, Montreal, Canada, between January 2012 and December 2014. Patients were selected through local pancreatic cancer databases from both centers. Eligible patients met the following criteria: age >18 years, confirmed histopathological PDAC, and received at least 1 cycle of FOLFIRINOX regimen in the first-line (FFX L1), followed by at least 1 cycle of gemcitabine monotherapy as second-line (GEM L2) treatment. Data collected were demographics, date of diagnosis, tumor characteristics, dates of start and discontinuation for FFX L1 and GEM L2, response rate (defined as the best response observed in each FFX L1 and GEM L2 regimen), date of progression on FFX L1 and on GEM L2, toxicities for both regimens, date of death or last visit, and cause of death.

### 2.2 Chemotherapy regimens

One cycle of FOLFIRINOX consisted of a combination of oxaliplatin (85 mg/m^2^), irinotecan (180 mg/m^2^), leucovorin, and 5FU (bolus and continuous infusion) as previously described.^[4]^. This cycle was repeated every 2 weeks until progression, death, unacceptable toxicity, or patient refusal. Primary or secondary prophylaxis of neutropenia using granulocyte colony-stimulating factor (G-CSF) was initiated at the physician's discretion. A 5FU bolus could be cancelled and oxaliplatin/irinotecan doses could be reduced if required. Gemcitabine, at a dose of 1000 mg/m^2^ was weekly delivered for 3 weeks in subsequent 4-week courses, until progression/death, unacceptable toxicity, or patient refusal. One cycle corresponded to a 4-week period of treatment. Further chemotherapy was recorded if given.

### 2.3 Toxicities

Toxicities were evaluated before each cycle of chemotherapy using the National Cancer Institute Common Terminology Criteria for Adverse Events (version 3.0).

### 2.4 Responses rates

Patients’ assessments during the GEM L2 regimen included computed tomography scan (CT scan) imaging after 2, 4, and 6 months of treatment and evaluation of the subjective clinical benefits. Tumor response was determined based on a thoracic-abdominal-pelvic CT scan according to the response evaluation criteria in solid tumor (RECIST criteria) version 1.1 and defined as follows: partial response (PR) if ≥30% decrease in the sum of the longest diameter of target lesions; progressive disease (PD) if ≥20% increase in this sum or appearance of new lesions, and stable disease (SD) in all other cases.

### 2.5 Statistical analysis

Demographic and clinical characteristics are shown as medians or frequencies, as appropriate. Progression-free survival 1 (PFS1) and progression-free survival 2 (PFS2) were defined from the first infusion of FFX L1 or GEM L2, respectively, until the date of disease progression based on imaging studies, discontinuation for toxicities, or death for GEM L2.

“Fast progressers” were defined as patients that received only 2 cycles of FFX L1 and/or 1 cycle of GEM L2 because of disease progression. OS was defined from the date of diagnosis until the date of death or last follow-up. SPSS version 10 (IBM, SPSS statistics, v10.0) was used for descriptive analysis, and survival curves were estimated using the Kaplan–Meier method.

Paoli-Calmettes institutional review board approved the study.

## 3 Results

### 3.1 Patients’ characteristics

We included 72 patients with advanced PDAC who received FFX L1 followed by GEM L2 (representing 55% and 60% of patients treated with FFX L1 for advanced PDAC in the Canadian and French databases, respectively). No statistical differences were observed between the Canadian and French patients in terms of demographics and treatment characteristics (data not shown); therefore, they were pooled all together for analysis.

The patients’ characteristics are shown in Table 1. The median age was 63.5 years (range, 32–75 years), and 9 patients (14%) were older than 70 years. The majority of the patients were men (n = 45, 62%). Four patients had a family history of women gynecological cancer (breast and/or ovarian cancers), none had a familial history of pancreatic cancer. The tumor location was predominantly the head of the pancreas (n = 37, 51%); 22 patients (30%) required biliary drainage at the diagnosis. At the time of diagnosis, 26 patients (36%) had locally advanced disease and 46 (64%) were metastatic. At initiation of GEM L2, 8 (11%) had locally advanced tumors, while 64 (89%) were metastatic. Metastases predominantly occurred in the liver (54%). In 11 patients (15%), the FFX regimen was switched to gemcitabine due to severe toxicity (neuroparesthesia, n = 7, severe diarrhea, n = 3 and vomiting, n = 1); in the remaining 61 patients, this was due to disease progression. All patients received FFX L1, and the median duration of FFX L1 was 4.8 months (ranging from 0.3 to 18 months); the overall response rate was 40% (29/72); most of the partial responses (25 of 29) were observed within the first 2 months. Conversely, at 2 months, 20 patients (28%) showed progression, including 2 fast progressers who progressed after 1 and 2 cycles of FFX L1.

**Table 1:**
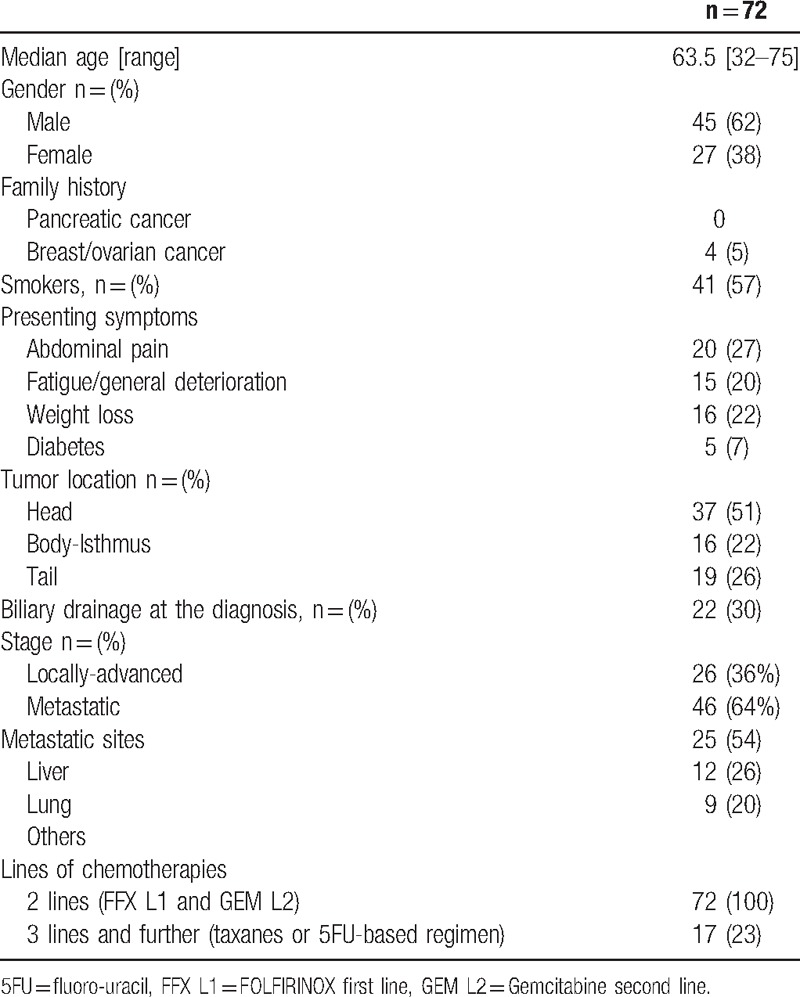
Patients’ characteristics.

Seventeen patients (23%) received third-line chemotherapies, including taxanes (paclitaxel and docetaxel monotherapies, n = 7), capecitabine (n = 4), or 5FU-based chemotherapies (FUFOL or FOLFOX, n = 6).

### 3.2 Results of GEM L2

The best overall responses on FFX L1 and GEM L2 are depicted in Table 2. The median duration of GEM L2 was 2.6 months (0.25–10.8), and it ranged from 1 to 8 cycles (median = 3 cycles). The objective response rate was 8/72 (11%), and disease control was observed in 25 patients (35%). Among the 11 patients that discontinued FFX L1 for toxicities (but stable disease on CT scan), 3 had objective responses with PR on GEM L2 after 2 months. In the 61 patients who progressed while on FFX L1, 5 obtained a PR (objective response rate of 8.2%). Among the 8 patients with PR after 2 months of gemcitabine, 4 (50%) still had PR after 4 months and 2 (25%) after 6 months of GEM L2. Among the 17 patients with SD after 2 months, 8 were stable after 6 months of GEM L2. Seven of these 17 patients (41%) had an early progression after 2 months on FFX L1. Four (5%) fast progressers were observed on GEM L2, and they only received 2 cycles of treatment before documentation of progressive disease. Less severe toxicities were observed during the GEM L2 regimen than those during FFX L1 regimen, and primarily included effusions (pleural and pericardial for 2 patients), edema (15%), and fatigue (18%). GEM L2 was discontinued due to major toxicities (fatigue) in 2 cases (2%). No treatment-related death was reported.

**Table 2:**
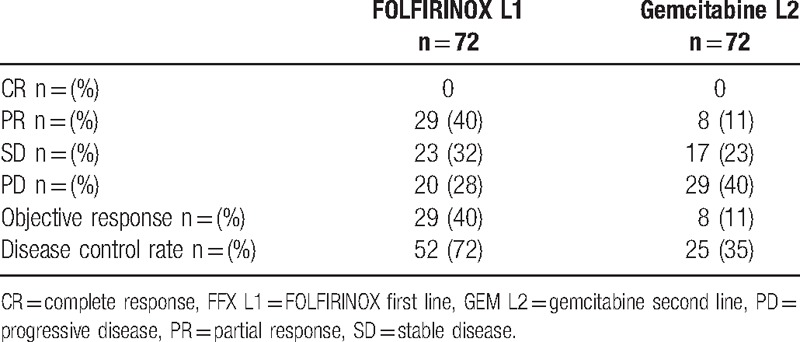
Best overall responses on FFX L1 and GEM L2.

### 3.3 Subjective clinical benefits with GEM L2

Subjective clinical benefits, including improvement in pain, general status and weight gain without edema, were documented in 24 of the 25 patients (96%) with disease control after 2 months of GEM L2. Among the 29 patients experiencing progressive disease under GEM L2 after 2 months, 8 patients (27%) mentioned a clinical benefit and improvement in their quality of life.

### 3.4 Survival

The median follow-up time was 18.3 months. The median OS for the entire cohort was 13.6 months (95% confidence interval [CI]: 2.0–35). The median PFS1 (FFX L1 regimen) was 4.8 months (95% CI: 0.4–18.4), and the median PFS2 (GEM L2 regimen) was 2.5 months (95% CI: 0.2–10.8).

**Figure 1:**
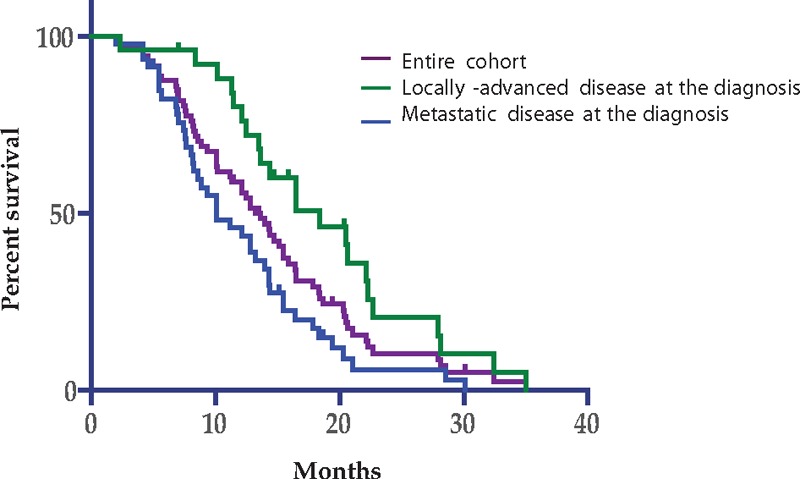
Overall survival.

The locally-advanced patient population at the time of diagnosis (n = 26) had a better OS of 18.4 months (95% CI: 7.0–35.0) and PFS1 of 6.2 months (95% CI: 0.4–14.4) compared with the metastatic patient population at the time of diagnosis (n = 46) with an OS of 10.1 months (CI 95%: 2.3–30.1) (*P* < .01) and PFS1 of 4.0 months (95% CI: 0.4–18.4) (*P* < .04) (Fig. 1). However, PFS2 was similar for the 2 groups: 2.6 months (95% CI: 0.2–10) and 2.3 months (95% CI: 0.2–15.1), respectively, (*P* = .47).

**Figure 2:**
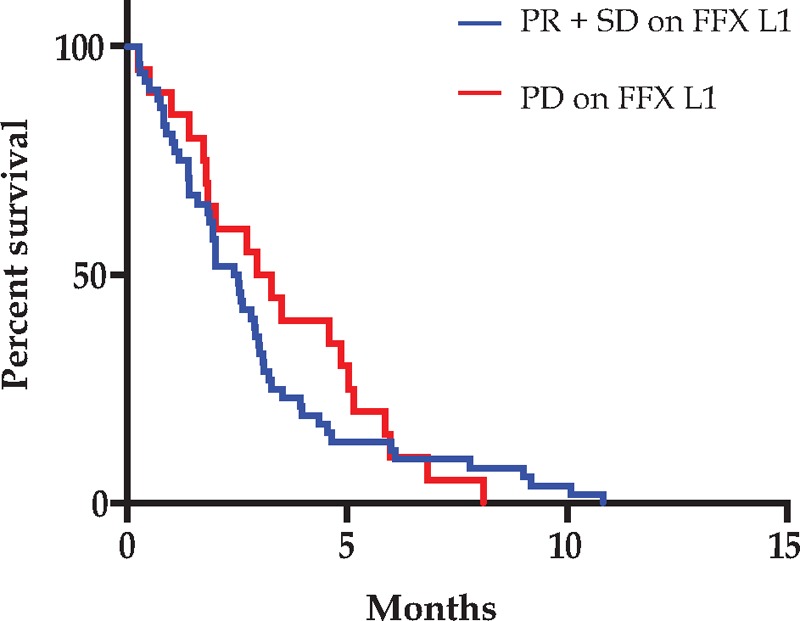
Progression free survival on GEM L2. T0 is defined as the time of initiation of gemcitabine. FFX L1 = FOLFIRINOX first line, GEM L2 = gemcitabine second line, PD = progressive disease, PR = partial response, SD = stable disease.

Similarly, PFS2 on gemcitabine was not significantly different between patients with controlled disease (n = 52) or progressers (n = 20) on FFX-L1 at 2 months, with a median PFS2 of 2.4 months and 3.1 months, respectively, but median OS was shorter for patients presenting with progressive disease (n = 20) after 2 months of FFX L1 with a median OS of 8.6 months (95% CI: 2.3–30.1) compared with those that had controlled disease with a median OS of 14.3 months (95% CI: 4.1–35; *P* = .02) (Fig. 2).

## 4 Discussion

This retrospective study is focused on advanced pancreatic cancer patients treated with 2 lines of chemotherapy, first with a FOLFIRINOX regimen and then gemcitabine monotherapy, and the results highlight the efficacy of gemcitabine as a second-line chemotherapy. Unexpectedly, the results barely depended on the efficacy of FFX in first-line treatment.

One-third of our patients in this retrospective study had locally-advanced PDAC for which the treatment is still controversial.^[5,6]^ Chemotherapy alone and chemoradiotherapy (CRT) are regarded as acceptable treatment options.^[7,8]^ For metastatic disease, there are currently 2 options for chemotherapy regimens that can be considered for first-line therapy in patients with good performance status, based on phase III trials: the FOLFIRINOX regimen^[4]^ and the combination of nab-paclitaxel + gemcitabine.^[9]^ Because nab-paclitaxel was not yet accepted in France and Canada, FOLFIRINOX was the first-line regimen given to patients with good performance status and under the age of 75 years. In our study, tumor response, PFS, and OS of patients treated with first-line FOLFIRINOX were consistent with but slightly better than those in the literature due to a selection bias (patients able to receive at least 2 types of treatment). Only 50% to 60% of all patients diagnosed with pancreatic cancer who fail first-line treatment are still sufficiently physically fit to be offered second-line treatment.^[10]^ A phase III trial compared, in second line post-gemcitabine, best supportive care (BSC) to “active” treatments and showed that cytotoxic agents given in second-line treatment improved OS.^[11]^ Whether gemcitabine as a second-line treatment is an appropriate choice after FOLFIRINOX failure remains to be determined. Before the FOLFIRINOX era, a strategic randomized controlled trial^[12]^ compared a well-tolerated combination of 5FU and CDDP (LV5FU-CDDP regimen) followed by gemcitabine versus gemcitabine followed by LV5FU-CDDP. This trial failed to demonstrate any advantage (survival, toxicity) of using the combination in the first-line treatment, and the study concluded that gemcitabine must remain the first-line treatment; in this trial, second-line gemcitabine gave an objective response rate of 7%. In this study, we reported our experience with gemcitabine in patients who failed after FOLFIRINOX, and we showed that gemcitabine can provide a median PFS of 2.5 months without major toxicity. Interestingly, some of the patients (8.2%) who previously experienced progressive disease on FFX L1 responded to the second-line treatment with gemcitabine, and some of the patients that discontinued FFX L1 because of toxicities also showed an objective response (3 of 11) and disease control. Thus, because of the different antitumoral mechanisms and actions between drugs, patients with FOLFIRINOX resistance could remain sensitive to gemcitabine. Moreover, many of our patients mentioned a clinical benefit of receiving gemcitabine monotherapy, including low toxicity, less fatigue/pain, and improved quality of life. Gemcitabine is not only an efficient antitumoral agent, but it is also a well-tolerated and effective drug. Several retrospective analyses of nab-paclitaxel/gemcitabine as a second-line treatment and beyond for metastatic pancreatic cancer have been described, but they have not yet demonstrated the feasibility, toxicity, or efficacy of using this combination after first-line FOLFIRINOX treatment and should be considered on a case-by-case basis.^[13–15]^

In conclusion, there is currently no standard of care for locally advanced or metastatic pancreatic cancer that has progressed following FOLFIRINOX. Although there are potential options, there is no demonstrable benefit for any regimen, and treatment choice is generally an extrapolation from front-line studies. Our retrospective study indicates that gemcitabine as the second-line treatment for some patients, even those who were not sensitive to FOLFIRINOX, can be useful, and it also resulted in disease control and symptom management. However, despite the modest impact of gemcitabine in second-line treatment for some patients, the majority of the patients developed resistance to the molecule in a short period of time, which strongly suggests that new approaches are needed.
